# Supplementary figures and images for: Human Breast Milk microRNAs, Potential Players in the Regulation of Nervous System
Source: Nutrients. 2023 Jul 24;15(14):3284. doi: 10.3390/nu15143284 (PMC10384760; doi:10.3390/nu15143284)

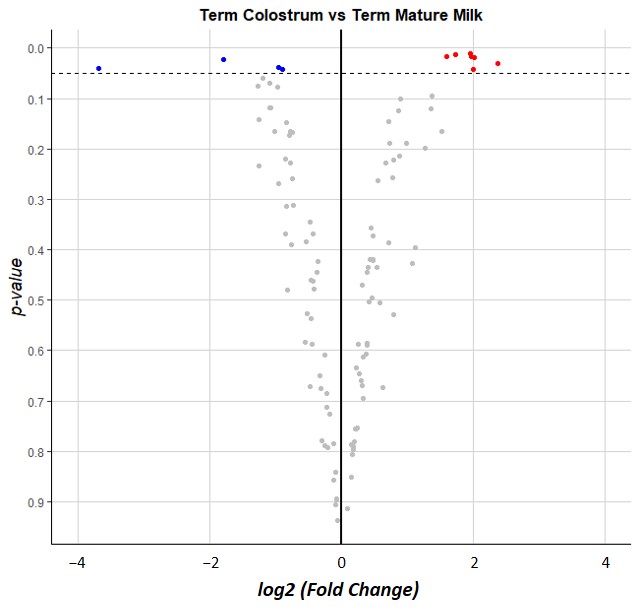

Supplement: Supplementary file 1 [file nutrients-15-03284-s001.zip › Supplemental Figure 1.jpg]

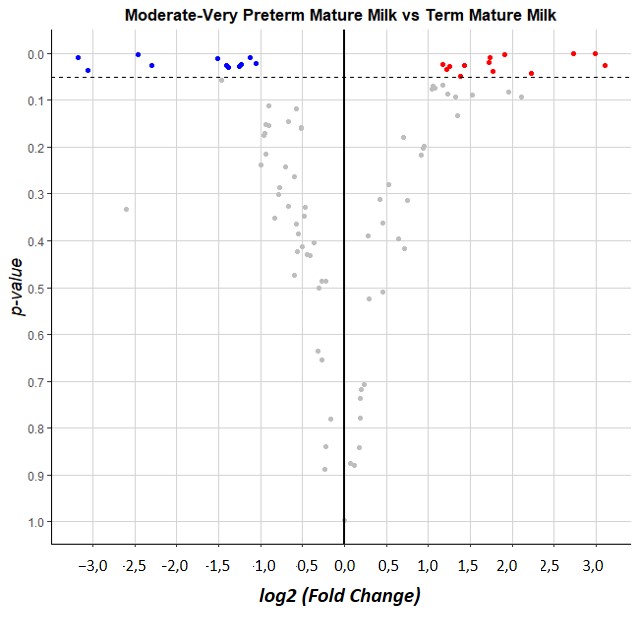

Supplement: Supplementary file 1 [file nutrients-15-03284-s001.zip › Supplemental Figure 2.jpg]

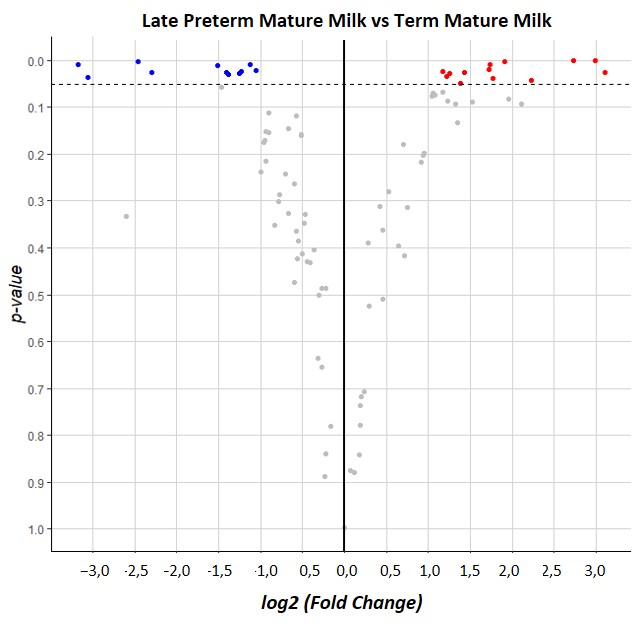

Supplement: Supplementary file 1 [file nutrients-15-03284-s001.zip › Supplemental Figure 3.jpg]

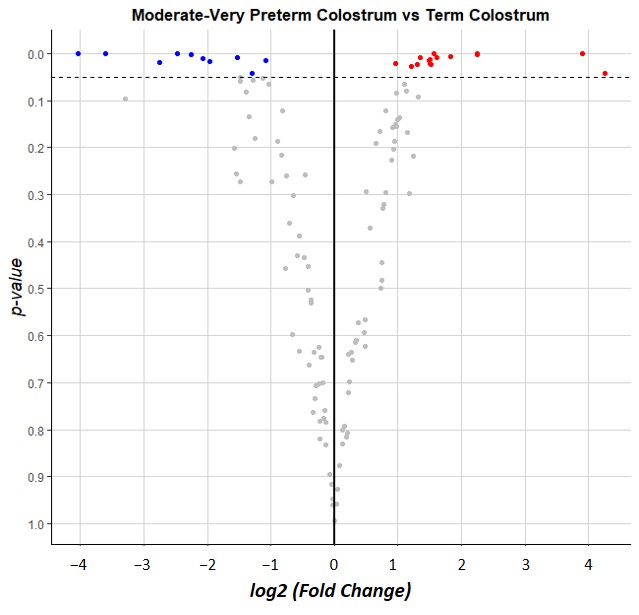

Supplement: Supplementary file 1 [file nutrients-15-03284-s001.zip › Supplemental Figure 4.jpg]

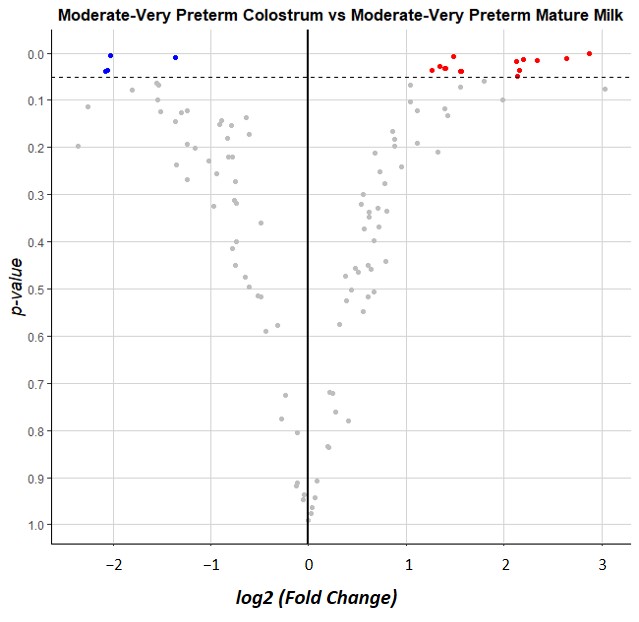

Supplement: Supplementary file 1 [file nutrients-15-03284-s001.zip › Supplemental Figure 5.jpg]

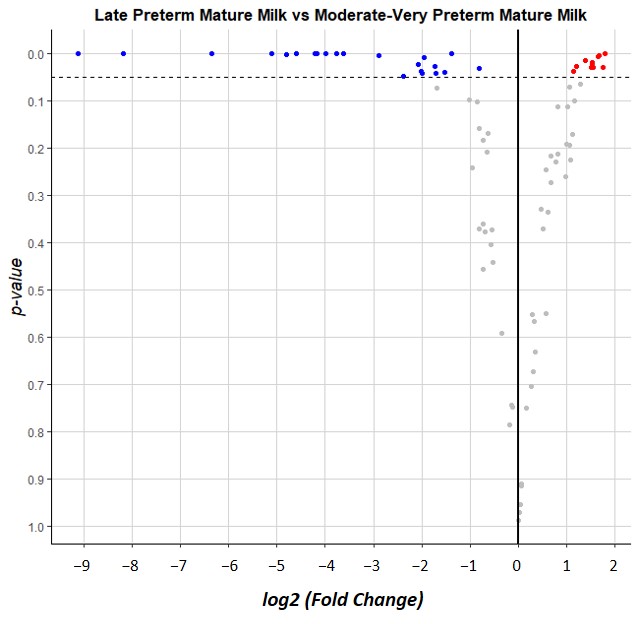

Supplement: Supplementary file 1 [file nutrients-15-03284-s001.zip › Supplemental Figure 6.jpg]
